# Increasing the economic value of lignocellulosic stillage through medium-chain fatty acid production

**DOI:** 10.1186/s13068-018-1193-x

**Published:** 2018-07-19

**Authors:** Matthew J. Scarborough, Griffin Lynch, Mitch Dickson, Mick McGee, Timothy J. Donohue, Daniel R. Noguera

**Affiliations:** 1 The Great Lakes Bioenergy Research Center, University of Wisconsin-Madison, Madison, WI USA; 2 Department of Civil and Environmental Engineering, University of Wisconsin-Madison, Madison, WI USA; 3 Department of Chemical and Biological Engineering, University of Wisconsin-Madison, Madison, WI USA; 4 Department of Bacteriology, University of Wisconsin-Madison, Madison, WI USA

**Keywords:** Medium-chain fatty acids, Mixed culture fermentation, Carboxylate platform, Chain elongation, Lignocellulose, Stillage

## Abstract

**Background:**

Lignocellulosic biomass is seen as an abundant renewable source of liquid fuels and chemicals that are currently derived from petroleum. When lignocellulosic biomass is used for ethanol production, the resulting liquid residue (stillage) contains large amounts of organic material that could be further transformed into recoverable bioproducts, thus enhancing the economics of the biorefinery.

**Results:**

Here we test the hypothesis that a bacterial community could transform the organics in stillage into valuable bioproducts. We demonstrate the ability of this microbiome to convert stillage organics into medium-chain fatty acids (MCFAs), identify the predominant community members, and perform a technoeconomic analysis of recovering MCFAs as co-products of ethanol production. Steady-state operation of a stillage-fed bioreactor showed that 18% of the organic matter in stillage was converted to MCFAs. Xylose and complex carbohydrates were the primary substrates transformed. During the MCFA production period, the five major genera represented more than 95% of the community, including *Lactobacillus*, *Roseburia*, *Atopobium*, *Olsenella*, and *Pseudoramibacter*. To assess the potential benefits of producing MCFAs from stillage, we modeled the economics of ethanol and MCFA co-production, at MCFA productivities observed during reactor operation.

**Conclusions:**

The analysis predicts that production of MCFAs, ethanol, and electricity could reduce the minimum ethanol selling price from $2.15 to $1.76 gal^−1^ ($2.68 gal^−1^ gasoline equivalents) when compared to a lignocellulosic biorefinery that produces only ethanol and electricity.

**Electronic supplementary material:**

The online version of this article (10.1186/s13068-018-1193-x) contains supplementary material, which is available to authorized users.

## Background

The production of food, fuels, pharmaceuticals and many chemicals depends on microbial fermentations. When one considers the sum of microbial biomass, excreted metabolic end-products, and non-metabolized nutrients, there is considerable residual organic matter in the liquid residue (stillage) remaining after distillation. One common co-product of ethanol production is biogas, which is generated by anaerobic digestion of stillage. Combusting lignin and biogas creates heat and power used to operate the biorefinery, and any excess electricity can be sold as a co-product [1]. In a techno-economic analysis (TEA) conducted by the National Renewable Energy Laboratory (NREL), a 61 million gallon per year lignocellulosic ethanol biorefinery produced fuel at a price of $2.15 gal^−1^ ($3.27 gal^−1^ gasoline-equivalents) and electricity worth $6.57 million year^−1^ [2].

The Renewable Fuel Standards (RFS), created by the Energy Policy Act of 2005 and expanded by the Energy Independence and Security Act of 2007, set production goals for many renewable energy sources, including lignocellulosic-derived ethanol [3, 4]. While several lignocellulosic biorefineries have been opened, total lignocellulosic ethanol production in the United States remains short of original targets. The high costs of obtaining biomass and producing enzymes to hydrolyze biomass are cited as barriers to achieving an acceptable level of profitability for lignocellulosic biorefineries [2].

One way to potentially improve the economics of lignocellulosic fuel production is to produce valuable co-products, such as medium-chain fatty acid (MCFA), from stillage. MCFAs are monocarboxylic acids containing six to twelve carbon atoms and are utilized for the production of rubbers, dyes, pharmaceuticals, and antimicrobials [5]. They can also be used as precursors for chemicals currently derived from fossil fuels [6]. In addition to being valuable, MCFAs also have decreased solubility compared to short-chain fatty acids (SCFA), which should allow for easier extraction from an aqueous medium.

In this study, we investigated the valorization of switchgrass-derived stillage to MCFAs. Switchgrass has been identified as a promising feedstock for biofuel production that can be cultivated on marginal lands [7]. In this study, we tested the ability of using mixed culture anaerobic fermentation, as in the so-called carboxylate platform [8, 9], to valorize stillage to MCFAs. Here MCFA is the sum of hexanoate and octanoate since it is still largely unknown how to direct metabolism to production of only one MCFA. In several past studies, ethanol has been utilized as an electron donor to drive MCFA production from either added acetate or acetate produced by the community as a fermentation intermediate [10–13]. Conversion of lactic acid to MCFA has also been investigated [14, 15]. Recently, a pure culture of *Megasphaera elsdenii* was used to convert glucose in lignocellulosic hydrolysate to MCFAs [16]. Stillage from corn-derived ethanol has also been used to produce MCFAs [17]. Andersen et al. utilized a mixture of lignocellulosic stillage and dilute ethanol to produce MCFAs at titers greater than their solubility concentrations [18]. However, MCFA production from industrial streams having minimal amounts of glucose or ethanol remains largely unexplored. In addition, there is no published TEA investigating production of MCFAs from stillage.

**Fig. 1:**
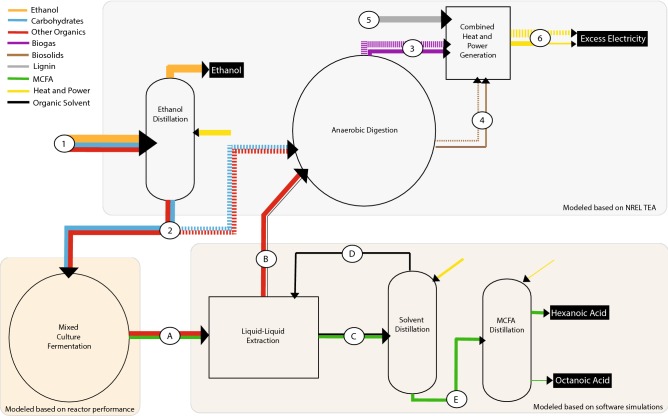
Energy balance through a proposed lignocellulosic biorefinery that simultaneously produces ethanol, electricity, and MCFAs. For this study, we modeled anaerobic digestion and combined heat and power generation using criteria used in a 2011 technoeconomic analysis performed by NREL. We modeled a MCFA-producing mixed culture fermentation process based on productivities observed in this study. Commercially available software was utilized for the modeling of downstream processes to extract and separate hexanoic and octanoic acids. In the proposed biorefinery, a portion of organic matter remaining in stillage is converted to MCFAs, which can be recovered via liquid–liquid extraction and distillation. Remaining organic matter undergoes anaerobic digestion to produce biogas that is combusted, along with lignin and biosolids, to produce electricity. Dashed lines represent current process flows for converting organic materials in stillage directly to biogas. Solid lines represent proposed processes for producing MCFA as a co-product. Line weights are proportional to approximate energy content represented as chemical oxygen demand (COD) for liquid and gas streams and as megawatts (MW) for heat and power. Process streams include (1) fermented hydrolysate; (2) stillage; (3) biogas; (4) biosolids; (5) lignin; (6) heat and power; (A) fermented stillage; (B) aqueous phase; (C) organic phase; (D) organic solvent; and (E) MCFAs

While past studies have investigated MCFA production from lignocellulosic materials, none have evaluated production of ethanol followed by MCFA production from the resulting stillage in a biorefinery. Thus, the objectives of this study are to (1) test the hypothesis that a stillage-fed microbial community can sustain production of MCFAs; (2) investigate the stability of the microbiome and potential roles of abundant community members in the MCFA-producing reactor; and (3) evaluate the technoeconomics of producing MCFAs from ethanol stillage. To achieve the third objective, we modeled a modified lignocellulosic biorefinery producing MCFA as a co-product of ethanol and electricity (Fig. 1). After accounting for the amount of organic matter in stillage that is directed to MCFA production, the reduction in overall biogas and electricity production, and the increased capital and operational costs associated with MCFA production, our data predict that the potential revenue from producing MCFAs at levels observed in this study would have a positive impact on the economics of lignocellulosic biorefining.

## Methods

### Switchgrass stillage production

Shawnee switchgrass, grown in 2010 at the Arlington Agricultural Research Center in Wisconsin, USA, was used as the biomass source for this study. Switchgrass was treated using ammonia-fiber expansion (AFEX), enzymatically hydrolyzed, and fermented, as described previously [19]. During processing, hydrolysate is filtered to remove insoluble components, including insoluble lignin. Past work has demonstrated that switchgrass hydrolysates generated with this process contain sufficient nutrients and trace elements to sustain microbial growth [19]. Ethanol fermentations of switchgrass hydrolysate were performed with *Saccharomyces cerevisiae* Y128, an engineered yeast strain with improved xylose utilization and lignotoxin tolerance [20]. Ethanol was removed post-fermentation using a glass distillation apparatus consisting of a 1-L boiling flask, heating mantle, distillation column, and condenser. During distillation, the fermented hydrolysate was heated to approximately 100 °C to maintain a distillation neck temperature of 78 °C. Therefore, the distillation process not only removed ethanol but also sterilized the stillage. The stillage remaining after distillation was stored at 4 **°**C until fed to the bioreactor.

### Mixed culture fermentation bioreactor

A mixed culture fermentation bioreactor was inoculated with sludge from an acid-phase digester at the Nine Springs Wastewater Treatment Plant in Madison, Wisconsin. The bench-scale reactor consisted of a vessel with a 150-mL working volume that was continuously stirred at 150 rpm with a magnetic stir bar and maintained at 35 °C using a water bath. The reactor was sealed with a rubber stopper and vented so that any gas produced was released to the atmosphere. For all experiments, the solid retention time (SRT) is equal to the hydraulic retention time.

Initially, we conducted short-term (6 day) experiments to assess if microbial growth could be sustained in stillage and to determine the primary fermentation end products under different pH conditions. For these initial experiments, the pH was either uncontrolled or controlled at set points of 5.0, 5.5, 6.0, or 6.5 with 5 M KOH. A hydraulic retention time of 2 days was utilized for these initial experiments by pumping 75 mL day^−1^ (3.13 mL h^−1^) both into and out of the reactor. A shorter SRT was utilized to allow for fast turnover and stabilization of the microbial community. While this short SRT resulted in production of MCFAs, we elected to increase the SRT for a long-term experiment in an attempt to improve overall MCFA titers. For the long-term (252 day) sustained experiment, the pH was controlled at a set point of 5.50 with 5 M KOH and the SRT was controlled at 6 days by pumping 25 mL day^−1^ (1.04 mL h^−1^) into and out of the reactor.

### Chemical analyses

We collected samples from the reactor and stillage for chemical analyses. All samples were filtered using 0.22-μm syringe filters (ThermoFisher Scientific SLGP033RS, Waltham, MA, USA). Soluble chemical oxygen demand (COD) analysis was performed using High-Range COD Digestion Vials (Hach 2125915, Loveland, CO, USA) as per standard methods [21]. Soluble carbohydrates were measured with the anthrone method [22]. Total soluble proteins were measured with the bicinchoninic acid assay using the Pierce™ BCA Assay Kit (ThermoFisher Scientific 23225, Waltham, MA, USA) and the Compat-Able™ Protein Assay Preparation Reagent Set (ThermoFisher Scientific 23215, Waltham, MA, USA) [23].

Glucose, xylose, acetic acid, formic acid, lactic acid, succinic acid, pyruvic acid, glycerol and xylitol were analyzed with high-performance liquid chromatography (HPLC) and quantified with an Agilent 1260 Infinity refractive index detector (Agilent Technologies, Inc. Palo Alto, CA) using a 300 × 7.8 mm Bio-Rad Aminex HPX-87H column with Cation-H guard (BioRad, Inc., Hercules, CA). A column temperature of 50 °C was used and 0.02 N H_2_SO_4_ was used for the mobile phase with a flow rate of 0.50 mL min^−1^.

Acetamide, ethanol, *n*-propionic acid, *n*-butyric acid, *iso*-butyric acid, *n*-pentanoic acid, *iso*-pentanoic acid, *n*-hexanoic acid, *iso*-hexanoic acid, *n*-heptanoic acid, and *n*-octanoic acid were analyzed with tandem gas chromatography–mass spectrometry (GC–MS). An Agilent 7890A GC system (Agilent Technologies, Inc. Palo Alto, CA) with a 0.25-mm Restek Stabilwax DA 30 column (Restek 11008, Belefonte, PA) was used. The GC–MS system was equipped with a Gerstel MPS2 (Gerstel, Inc. Baltimore, MD) autosampler and a solid-phase micro-extraction gray hub fiber assembly (Supelco, Bellefonte, PA). The MS detector was a Pegasus 4D TOF–MS (Leco Corp., Saint Joseph, MI). Stable isotope-labeled internal standards were used for each of the analytes measured with GC–MS.

Aromatic compounds were analyzed with liquid chromatography–tandem mass spectrophotometry (LC–MS/MS). For LC–MS/MS analyses, an Ultimate HPG-3400RS pump and WPS-3000RS autosampler (ThermoFisher) were mated to an ACQUITY UPLC HSS T3 reversed-phase column (2.1 × 150 mm, 1.8 μm particle diameter, Waters Corporation) with a guard cartridge. Gradient elution was performed at 0.400 mL min^−1^. The LC system was coupled to a TSQ Quantiva Triple Quadrupole mass spectrometer (Thermo Scientific). The Ion Transfer Tub Temp was kept at 350 °C as was the vaporizer temperature. Analytes measured with LC–MS/MS included vanillamide, 4-hydroxybenzyl alcohol, syringamide, coumaryl amide, 4-hydroxybenzoic acid, feruloyl amide, vanillic acid, *p*-coumaric acid, ferulic acid, and benzoic acid. Detailed chemical analysis data are provided in Additional file 1.

### Microbial community analysis

Amplification and sequencing of the V3–V4 region of the 16S rRNA gene were performed to classify and determine the relative abundance of bacteria in the reactor. For the initial short-term (6 day) experiments, biomass samples were collected from the inoculum acid digester sludge and from the reactor every 2 days for 6 days. For the long-term (252 day) experiment, biomass samples were collected from the inoculum acid digester sludge and from the reactor at days 2, 4, and 6, and then every 6 days for the duration of the experiment. Biomass was harvested by centrifuging samples at a relative centrifugal force of 10,000*g* for 10 min and decanting supernatant. Biomass was then stored at − 80 °C until DNA extraction was performed.

DNA was extracted using a Power Soil^®^ DNA Isolation Kit (MoBIO Laboratories 12888, Carlsbad, CA). The purity of extracted DNA was analyzed using a NanoDrop spectrophotometer (Thermo Fisher Scientific ND-2000, Waltham, MA), and DNA was quantified using a Qubit 3.0 (Thermo Fisher Scientific Q33126, Waltham, MA). The V3 and V4 regions of the 16S rRNA gene were amplified using the primer set S-D-Bact-0341-b-S-17/S-D-Bact-1061-a-A-17 as described by Klindworth et al. [24]. Amplicons were sequenced on an Illumina MiSeq sequencer (Illumina, San Diego, CA) using pair-end 250 base pair kits at the University of Wisconsin-Madison Biotechnology Center.

Paired-end reads were merged with Fast Length Adjustment of Short Reads (FLASH) using default parameters [25]. The merged reads were analyzed with the Qiime pipeline, utilizing the split libraries command to remove low-quality sequences [26]. Sequences were clustered into operational taxonomic units (OTUs) using uclust [27]. Sequences were aligned with PyNast, and chimera detection was performed with ChimeraSlayer [28, 29]. Singleton OTUs were removed, and the samples were rarefied to an equal depth, with 130,000 sequences retained for the long-term (252 day) reactor experiment and 45,000 sequences retained for the short-term (6 day) reactor experiments. A representative sequence for each OTU was taxonomically classified using the SILVA database [30]. Tables of OTUs with taxonomic assignments are provided in Additional file 2. The Phyloseq package version 1.14.0 was used for data visualization and heat maps were generated with the superheat package [31, 32]. To construct phylogenetic trees, multiple sequence alignments were performed using MUSCLE, and maximum-likelihood phylogenetic trees were constructed with RAxML using the GTRGAMMA method with 1000 bootstraps [33, 34].

Statistical analysis of microbial community data was performed using multivariate repeated measures ANOVA with the *nlme* package in R to generate generalized least square models in which time was correlated to all predictor variables using the corAR1 structure [35]. Redundancy analysis was also performed using the *rda* command in the vegan package [36]. Environmental factors were iteratively selected until all were statistically significant (p < 0.1) based on 999 model permutations.

### Technoeconomic analysis

To estimate the economic impact of producing MCFAs from ethanol stillage, a TEA was performed based on information provided in the National Renewable Energy Laboratory (NREL) TEA for a 61 million gallon per year lignocellulosic ethanol facility [2]. We assumed that switchgrass has a similar feedstock cost to corn stover ($58.50 U.S. dry ton^−1^), which is within the range of costs assumed for switchgrass feedstock in other studies [37, 38]. Instead of assuming all stillage undergoes anaerobic digestion, we assumed that a portion of the organic matter was converted to organic acids using data obtained in this study and then simulated the extraction of hexanoic and octanoic acids with ASPEN (AspenTech, Bedford, MA) to select an organic solvent and determine process separation efficiencies, heating demands, and sizes for reactors and equipment. We selected 2-octanol as the solvent for liquid–liquid extraction due to the high extraction efficiencies predicted with ASPEN. We assumed that the organic matter in the aqueous phase that remains after extracting the MCFAs was fed to the anaerobic digester to produce biogas. The specific methane yield (g methane produced per g COD consumed) and biosolid yield (g biomass produced per g COD consumed) were assumed to be the same as in the NREL TEA [2]. The efficiency of combined heat and power generation by combusting biogas, lignin, and biosolids was also assumed to have the same efficiency as the NREL TEA, with a total of 21% of the energy in the combusted material converted to usable heat and power [2].

The costs for additional reactors and distillation columns were estimated by scaling-related costs presented in the NREL TEA [2]. Costs for the liquid–liquid extraction were determined based on the volumetric flow rate and equations available in Seider et al. [39]. The KOH usage was calculated based on experimental reactor data. The 2-octanol demand (2-octanol lost to the aqueous phase) was based on modeling the liquid–liquid extraction with ASPEN. Prices for hexanoic acid, octanoic acid and 2-octanol were obtained from Zauba for imported quantities greater than 1000 kg in 2016 (Additional file 3) [40]. For consistency with past reporting, all costs and profits are reported in 2007 United States Dollars (USD). To convert from 2016 to 2007 USD, cost indices from the St. Louis Federal Reserve were used [41]. Electricity prices from the NREL TEA were used [2]. A 30-year cash flow was calculated using the cash-flow calculation tool available with the NREL TEA [42], and the minimum ethanol selling price (MESP) was determined by setting the net present value to zero based on a target 10% internal rate of return, consistent with the NREL TEA [2]. Detailed information related to the TEA is provided in Additional file 3.

### COD calculations

Unless otherwise noted, we report concentrations as mass of COD per unit volume. This allows for the direct comparison of relative reducing equivalents contained within each of the compounds consumed and created. The theoretical COD of each compound, or the theoretical amount of oxygen needed to fully oxidize the compound, was used to convert the measured mass units to COD. Protein was assumed to have 1.5 g COD per g of protein, which is consistent with the COD of albumin. A COD of 1.06 g COD per g carbohydrate was used to convert total carbohydrates measured with the anthrone method to COD. This value is consistent with the COD of glucose and xylose. The “Unknown COD” represents the measured COD minus the COD of known components. Where provided, error bars represent standard deviation of technical replicates. The “COD Removed” is calculated as the percentage of COD removed at each time point. “Conversion of Carbohydrates” is calculated based on the difference between total carbohydrates in the switchgrass stillage and the reactor sample for each time point. “Conversion to SCFA” is based on the amount of COD converted to carboxylic acids containing two to five carbons, and “Conversion to MCFA” is based on the COD converted to monocarboxylic acids containing six to eight carbons.

## Results

### Chemical analyses of switchgrass stillage

In a lignocellulosic biorefinery, an ethanologenic microorganism ferments biomass sugars to ethanol and the ethanol is removed via distillation, producing an organic-rich stillage fraction. The concentrations of compounds remaining in stillage are, therefore, dependent on the efficiency of the upstream fermentation. For this study, two batches of stillage (Table 1) were produced from switchgrass hydrolysate fermented with *S. cerevisiae* Y128, a strain with improved utilization of xylose [20]. The starting glucose and xylose concentrations in the hydrolysate prior to fermentation were 56,000 ± 300 mg COD L^−1^ and 36,000 ± 200 mg COD L^−1^, respectively. After the fermentation, the ethanol concentration was 51,000 ± 2900 mg COD L^−1^ with nearly 100% of the glucose and 47% of the xylose consumed. Glycerol, a common byproduct of yeast fermentation [43], reached a final concentration of 2500 ± 100 mg COD L^−1^. Acetic and formic acids decreased slightly during the ethanologenic fermentation, and only a small amount of lactic acid (30 ± 1 mg COD L^−1^) was detected (Additional file 1: Table S1). The total COD of the two batches of fermented hydrolysate was 160,000 ± 1500 mg COD L^−1^ (Additional file 1: Table S2).

**Table 1:** Major chemical components contained within hydrolysate and fermented hydrolysate after fermentation with *Saccharomyces cerevisiae* Y128

	Hydrolysate	Fermented hydrolysate
Glucose	56,000 ± 300	44 ± 1.7
Xylose	36,000 ± 230	19,000 ± 4500
Glycerol	310 ± 0.86	2500 ± 130
Acetic acid	2065 ± 30	1600 ± 68
Ethanol	< 100	51,000 ± 2900

The COD remaining in stillage, after distilling ethanol from the fermented hydrolysate, was approximately 60% of the COD in the fermented hydrolysate. The major chemical energy components in the stillage included xylose, acetamide (derived from acetate during ammonia-based pretreatment of switchgrass), glycerol, and acetic acid (Table 2). Residual glucose was minimal (Table 1), and the ethanol that was not removed in distillation (Table 2) represented less than 3% of the ethanol present in the original fermentation broth (Table 1). Carbohydrates, excluding xylose, accounted for 18% of the COD, while proteins accounted for only 2.2% of the COD in the stillage. In addition, a large portion of the COD is comprised of components with undetermined chemical identity. This “Unknown COD” likely contains a variety of compounds that are either produced during biomass deconstruction, originate from the switchgrass, or are produced during the yeast ethanol fermentation.

**Table 2:** Composition of major organic matter components and aromatic compounds in the two batches of stillage fed to the mixed culture fermentation bioreactor

	Stillage batch 1	Stillage batch 2
Major stillage components (mg COD L^−1^)		
Soluble COD	95,400 ± 432	95,800 ± 982
Unknown COD	38,300 ± 3250	42,100 ± 3190
Xylose	20,800 ± 148	20,900 ± 168
Other carbohydrates	19,300 ± 2310	15,500 ± 2230
Acetamide	4030 ± 270	4200 ± 340
Glycerol	3900 ± 32.1	3920 ± 36.3
Acetic acid	2550 ± 21.1	2580 ± 20.5
Proteins	2200 ± 145	1910 ± 162
Ethanol	1220 ± 305	1590 ± 161
Aromatic compounds (μg COD L^−1^)		
Coumaroyl amide	13,000 ± 250	5400 ± 200
Feruloyl amide	12,000 ± 130	3200 ± 83
*p*-Coumaric acid	3500 ± 43	1100 ± 34
Benzoic acid	1700 ± 102	2000 ± 22
Vanillamide	290 ± 0.95	230 ± 0.50
4-Hydroxybenzoic acid	380 ± 15	320 ± 0.46
Vanillic acid	320 ± 0.09	370 ± 4.6
Ferulic acid	250 ± 13	90 ± 3.2
4-Hydroxybenzyl alcohol	240 ± 3.7	110 ± 1.9
Syringamide	230 ± 0.06	138 ± 2.3

Major stillage components are reported in mg COD L^−1^ whereas aromatic compounds are reported in μg COD L^−1^

While major COD components between the two batches of stillage were similar, the aromatic compounds, including known lignotoxins [19, 44], varied between the stillage batches (Table 2). Feruloyl amide, *p*-coumaroyl amide, and coumaric acid were higher in batch 1 than in batch 2. Only benzoic acid and vanillic acid were higher in batch 2. From a reducing-equivalent standpoint, these aromatic compounds account for less than 0.05% of the COD in stillage, but these concentrations are within the range of lignotoxins shown to inhibit fermentation activity by pure cultures of ethanologenic organisms [20].

### Stillage fermentation under different pH conditions

Due to the relatively low concentration of six-carbon sugars, the complexity of remaining organic materials, and the potential toxicity of aromatic compounds, bacterial growth on stillage derived from AFEX-treated hydrolysate was expected to be challenging. We, therefore, conducted short-term experiments to determine if a microbial community could metabolize organic materials remaining in stillage. Using inoculum from an acid-phase anaerobic digester, we fermented stillage at different pH conditions (uncontrolled, 5.0, 5.5, 6.0, and 6.5) for 6 days utilizing a SRT of 2 days and analyzed both the extracellular end products and the microbial community. Acid-phase digester sludge was used as inoculum because the microbial consortia were expected to contain a variety of fermenting organisms and not expected to contain high levels of methanogens [45, 46].

Conditions in which the pH was uncontrolled led to the pH stabilizing at 3.6 and accumulation of lactic and acetic acids (Additional file 4). SCFA accumulated in the reactor when the pH was maintained between 5.0 and 6.5. Maintaining a pH of 5.5 resulted in the highest accumulation of MCFAs (Additional file 4). Analysis of the microbial community by 16S rRNA gene sequencing showed variations in composition with pH (Additional file 5), with OTUs associated with the genera *Lactobacillus* (89.9%) and *Acetobacter* (9.9%) becoming the most abundant when the pH was uncontrolled. *Lactobacillus* was present in the reactors at all pH conditions. At pH 5.0, *Megasphaera* was enriched (46.3%), while at pH 5.5, OTUs related to *Pseudoramibacter* (14.3%) and *Olsenella* (14.1%) were abundant. At pH 6.0, *Mitsuokella* (20.8%), *Acetitomaculum* (17.0%), and *Megasphaera* (14.2%) were all abundant. When the reactor was maintained at pH 6.5, more OTUs related to the *Bacteroidetes* phylum were abundant, including OTUs related to the genera *Prevotella* (12.3%) and *Bacteroides* (40.8%).

These results demonstrated that a community derived from an acid digester sludge inoculum could ferment stillage to carboxylic acids, including MCFAs, under a variety of pH conditions. Further, organisms identified in the stillage-fed reactors included members of the *Clostridia (Megasphaera, Pseudoramibacter)* that have previously been associated with MCFA production [5, 10, 13, 15, 18, 47]. Members of *Clostridia* have been enriched in other MCFA-producing bioreactors under similar pH conditions [12, 18, 48]. In agreement with our observation of *Lactobacillus* at all pH conditions, *Lactobacillus* is a common genus in MCFA-producing microbiomes [10, 15, 17, 18, 47]. In total, the fermentation product (Additional file 4) and community (Additional file 5) data confirmed that materials in stillage could be converted to MCFAs by a microbial community originating from a full-scale wastewater treatment plant acid digester.

### Sustained MCFA production from switchgrass stillage

**Fig. 2:**
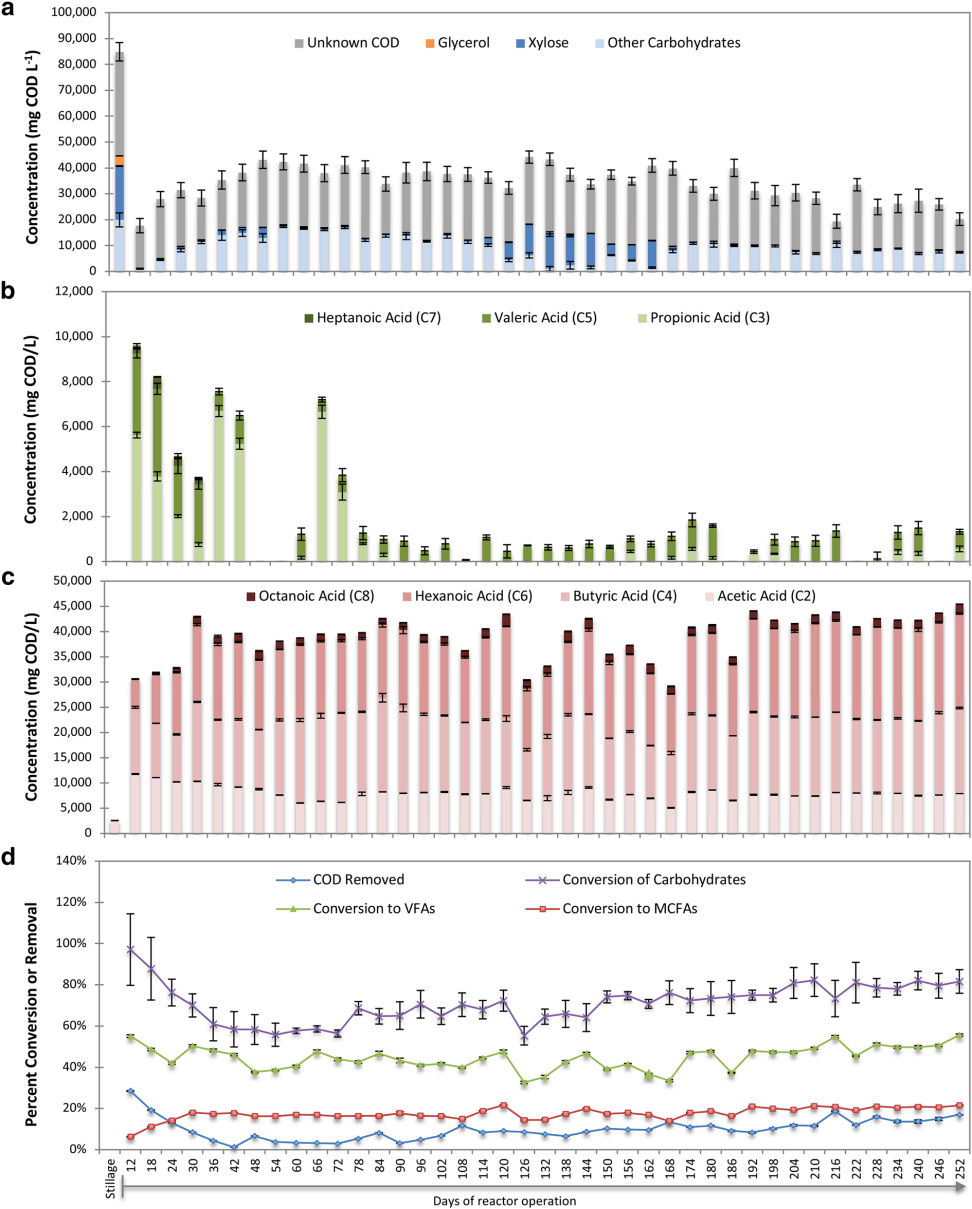
Mixed culture fermentation reactor performance for 252 days. **a** Compounds removed from stillage; **b** production of odd-chain propionic (C3), valeric (C5), and heptanoic (C7) acids; **c** production of even-chain acetic (C2), butyric (C4), hexanoic (C6), and octanoic (C8) acids; **d** removal of COD, percent conversion of carbohydrates, and percent conversions of COD to SCFA (C2–C5) and MCFAs (C6–C8)

Based on these results, we chose to control the reactor pH at 5.5 for a long-term experiment to demonstrate sustained production of MCFAs. Initially, xylose and other carbohydrates were consumed, and a mixture of odd- and even-chain linear fatty acids was produced (Fig. 2a–c). The maximum utilization of carbohydrates was achieved at Day 12, with 97 ± 17% of the measured initial carbohydrates consumed (Fig. 2d). During the first 30 days of operation, accumulation of monocarboxylic acids steadily increased, reaching nearly 50% conversion of COD in stillage to monocarboxylic acids (Fig. 2d). As reactor operation continued, the concentration of odd-chain monocarboxylic acids (C3, C5 and C7) decreased (Fig. 2b) while that of even-chain acids increased (Fig. 2c). From Day 30 through Day 252, the average conversion of COD in stillage to MCFAs was 18 ± 2.1%, and MCFAs accounted for 41 ± 7.0% of the total monocarboxylic acids produced.

### Microbial community analysis

**Fig. 3:**
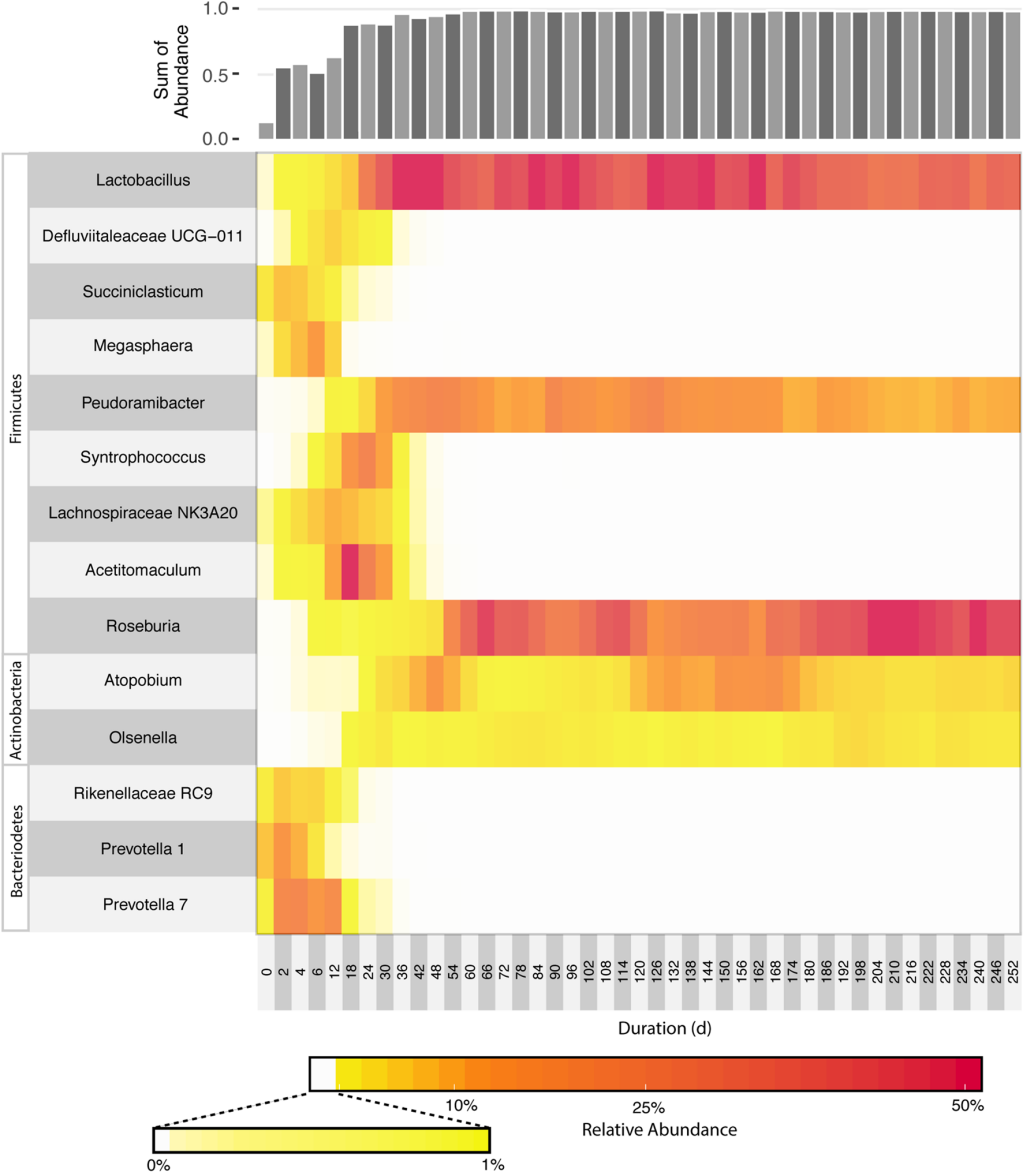
Relative abundance of bacteria in the mixed culture fermentation reactor for 252 days. Day 0 corresponds to the acid digester sludge inoculum. Bacterial abundance is summarized based on the genera assigned by annotating representative sequences with the SILVA database. The sum of abundance represents the percentage of OTUs contained within the indicated genera. A heat map of the top 100 OTUs is provided in Additional file 3: Figure S3 and a table of all OTUs are provided in Additional file 4: Table S1

We used 16S rRNA gene amplicon sequencing to assess the members of the microbial community in this bioreactor and any changes that occurred in its composition as a function of time (Fig. 3; Additional file 6). The initial microbial community contained many *Proteobacteria*, *Firmicutes*, and *Bacteroidetes* (Additional file 6). Early on in reactor operations, *Bacteroidetes* and *Firmicutes* became the most abundant organisms, with *Prevotella* species accounting for most of the *Bacteroidetes*. The increase in abundance of *Prevotella* 7 (Fig. 3) corresponds with the time of increased carbohydrate conversion (*p* < 0.001), in agreement with *Prevotella*’*s* described ability to degrade polysaccharides and other complex substrates [49]. *Megasphaera*, an organism known to produce odd-chain fatty acids (OCFA) [50], was present in the inoculum and increased in abundance during the early phase of reactor operation. The high abundance of *Megasphaera* (*p* = 0.0023) and *Prevotella* 7 (*p* = 0.0016) at early stages of reactor operation corresponded with a period of higher OCFA production.

After extended operation, we found that the community composition stabilized and was dominated by organisms from five genera, including three Firmicutes (*Lactobacillus*, *Pseudoramibacter* and *Roseburia*) and two Actinobacteria (*Olsenella* and *Atopobium*). At later time points (Day 30–Day 252), the OTUs corresponding to these five genera accounted for greater than 95% of the total 16S rRNA gene sequences (Fig. 3). The relative abundance of *Pseudoramibacter* (*p* = 0.0045), *Lactobacillus (p *= *0.0022)*, and *Olsenella (p *=* 0.014)* all correlated with the period of increased MCFA production. Neither *Roseburia* (*p* = 0.147) nor *Atopobium* (*p* = 0.546) are significantly correlated to increased MCFA production.

**Fig. 4:**
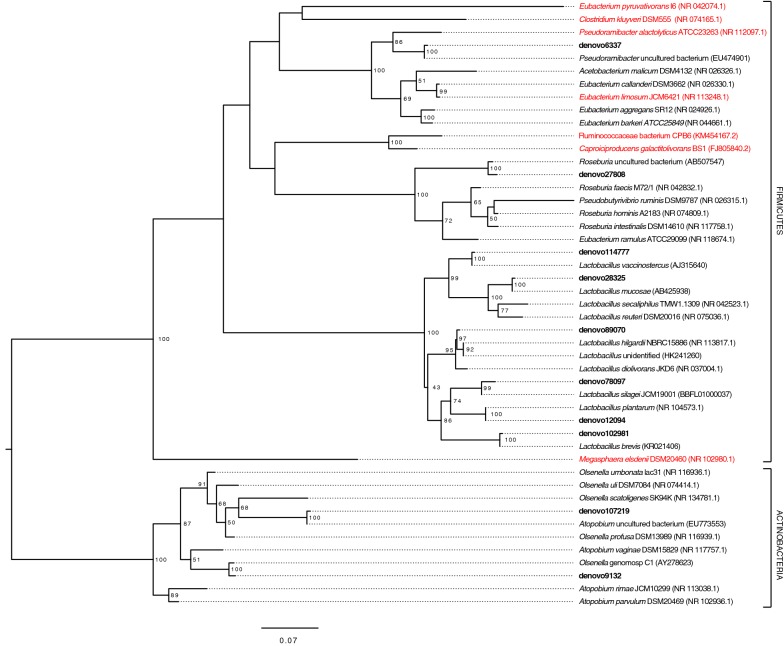
Phylogenetic tree including the top ten most abundant OTUs at Day 252. OTUs from this study are shown in bold text. Known chain-elongating bacteria are shown in red text. Bootstrap values greater than 50 are shown, and the phylogenetic tree is rooted to the Actinobacteria phylum. The horizontal branch distance corresponds to the mean nucleotide substitutions per sequence site. The accession numbers for 16S rRNA gene sequences for the indicated bacteria are provided in parentheses

**Fig. 5:**
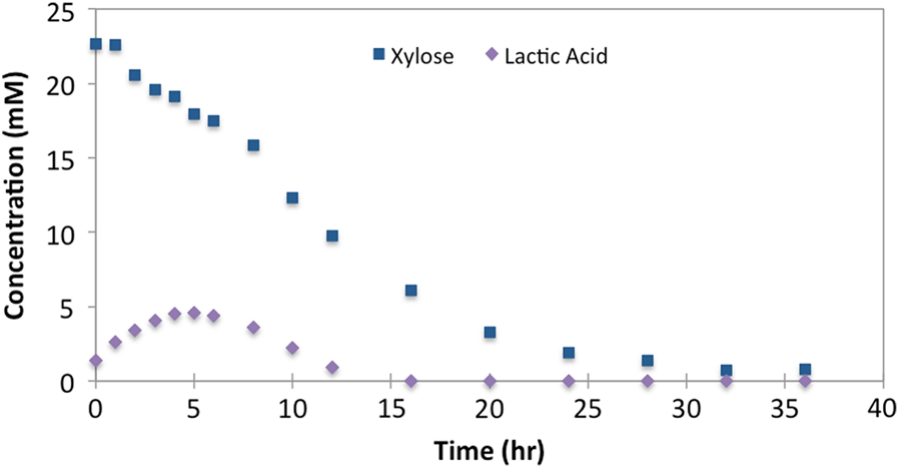
Time-dependent changes of xylose and lactic acid concentrations. Lactic and xylose were measured after adding a spike feed of 25 mL stillage to the reactor at 252 days of operation. As xylose is removed from the media, lactic acid accumulates at approximately 1 mol of lactic acid per mol of xylose consumed. Extracellular lactic acid begins to decrease 6 h after the addition of stillage

Representative sequences for the most abundant OTUs were used to construct a maximum-likelihood phylogenetic tree (Fig. 4). The six high-abundance *Lactobacillus* OTUs (denovo114777, denovo28325, denovo102981, denovo12094, denovo78097, and denovo89070) clustered with known xylose-consuming, heterofermentative Lactobacilli (*L. mucosae*, *L. plantarum*, *L. silagei*, *L. brevis*, *L. vaccinostercus* and *L. diolivorans*) [51–58]. As lactic acid has previously been demonstrated as a substrate for MCFA production [14, 15], it may be a key intermediate for MCFA production in a microbial community [18]. While significant lactic acid accumulation was not observed during steady-state sampling, when we monitored time-dependent changes in the reactor after stillage was spike-fed, lactic acid transiently accumulated to detectable levels in the medium (Fig. 5) suggesting that lactic acid is produced but consumed by other community members.

The two OTUs within the Actinobacteria phylum, denovo9132 and denovo107219, clustered with members of the *Atopobium* and *Olsenella* genera, respectively (Fig. 4), in the Coriobacteriaceae family. Several *Atopobium* and *Olsenella* species have been shown to consume carbohydrates and produce lactic acid [59–62]. The most abundant OTU at 252 days of reactor operation (denovo27808) clustered with *Roseburia*, which are known to utilize carbohydrates and acetic acid and produce butyric and lactic acids [63–65]. Another high-abundance OTU identified in the microbial community (denovo6337) clustered with *Pseudoramibacter alactolyticus* (previously *Eubacterium alactolyticum*), a bacterium that has been described as producing hexanoate and octanoate from lactic acid and glucose [66].

Starting at Day 120, the feed changed from stillage batch 1 to stillage batch 2, which contained lower concentrations of aromatic compounds (Table 2). While initial changes in community compositions occurred (Fig. 3), with an increase in *Atopobium* and decrease in *Roseburia*, the major genera remained consistent and the community eventually re-stabilized. This initial change in stillage feed source coincided with a reduction in xylose utilization (Fig. 2); however, xylose utilization eventually increased and overall MCFA production was not impacted by this change in the stillage source (*p* = 0.415).

**Fig. 6:**
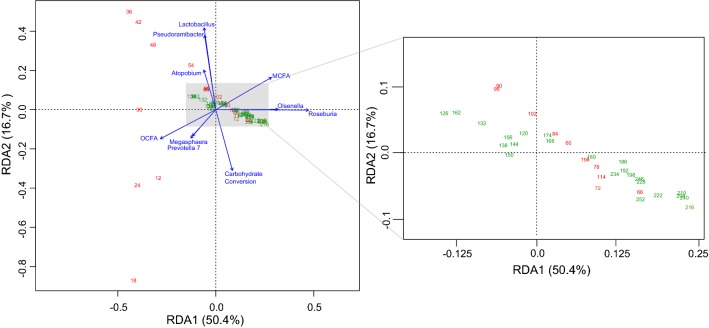
Redundancy analysis of microbial community and fermentation reactor performance. The percent variance explained by each axis is indicated in parentheses. The numbers within the plot indicate the day of sampling. Red numbers indicate time points in which stillage batch 1 was fed to the reactor, and green numbers indicate time points in which stillage batch 2 was fed to the reactor. Arrows indicate environmental factors related to community composition and the sum of individual genera within the bacterial community. These include the sum of propionic, valeric, and heptanoic acids (OCFA; *p* = 0.001), the sum of hexanoic, heptanoic, and octanoic acids (MCFAs; *p* = 0.001), and the total percentage of carbohydrates converted to fermentation products (carbohydrate conversion; *p* = 0.001)

We also performed redundancy analysis to relate the community composition with MCFA production, odd-chain fatty acid production (OCFA), and carbohydrate conversion and to investigate co-occurrence of abundant bacteria in the reactor (Fig. 6). For early time points (Days 12–24), the abundance of *Prevotella* and *Megasphaera* correlates with OCFA production. The analysis also showed that higher relative abundance of *Lactobacillus* is associated with higher relative abundance of *Pseudoramibacter* and higher relative abundance of *Roseburia* correlates with higher relative abundance of *Olsenella* (Fig. 6). These correlations suggest that these organisms may work in tandem during stillage metabolism. In the case of *Lactobacillus* and *Pseudoramibacter*, the Lactobacillus may be producing lactate that *Pseudoramibacter* converts to MCFAs. This relationship is analogous to that suggested by Andersen et al. in which *Megasphaera* utilized lactate generated by *Lactobacillus* [17]. Similarly, *Olsenella* may be producing intermediates, such as acetate, that are known to be utilized by *Roseburia*.

Overall, the bacterial community results indicate that a stable fermenting community containing only five genera was enriched from the acid-digester sludge inoculum during growth on stillage. We suspect that *Clostridia*-related organisms (*Pseudoramibacter* and/or *Roseburia*) are responsible for MCFA production and the remaining community members ferment sugars to intermediate compounds (acetate, lactate, and/or ethanol) that provide substrates for MCFA production.

### Economic analysis of MCFA production from stillage

Based on the sustained production of MCFAs in this study, we evaluated the potential value of this process. We did this by modifying the NREL TEA for a lignocellulosic ethanol biorefinery to include a process in which stillage is used to produce MCFAs. Using average percent conversions in the bioreactor between Day 30 and Day 252, we estimated that the COD remaining in stillage was converted to end products at the following percentages: 5.4% acetic acid, 15% butyric acid, 16% hexanoic acid, and 1.7% octanoic acid. Further, based on reactor operations during the same time period, 9.1% of the COD is removed from the system as off-gas.

**Fig. 7:**
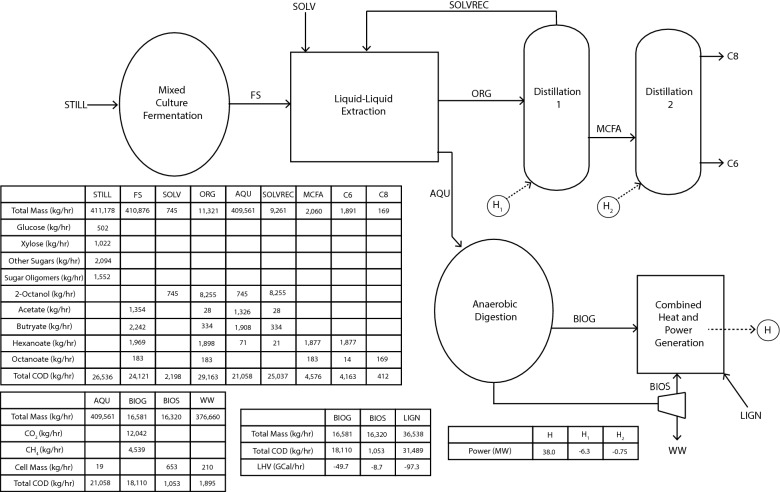
Proposed mass and energy balance for co-production of ethanol and MCFAs from lignocellulosic ethanol stillage. The following process flow indicators are used: *STILL* stillage, *FS* fermented stillage, *SOLV* organic solvent, *AQU* aqueous phase, *SOLVREC* recovered solvent, *BIOG* biogas, *BIOS* biosolids, *WW* wastewater, *LIGN* lignin, *H* generated heat and power, *H1* heating duty for distillation column 1, *H2* heating duty for distillation column 2

Based on these conversions, a new mass and energy balance for the biorefinery was determined (Fig. 7). The MCFA-producing fermentation reactor was sized for a SRT of 6 days, yielding an estimated total reactor volume of 16 million gallons. Software simulations predicted that a solvent flow rate of 9000 kg h^−1^ was needed to recover 99.9% of the octanoic acid and 96.4% of the hexanoic acid, respectively. Software simulations further predicted that of the 9000 kg h^−1^ 2-octanol feed, 745 kg h^−1^ separates into the aqueous phase and needs to be replenished. In our TEA, the organic phase undergoes a column distillation to remove 2-octanol that has a volume of 630 ft^3^ and requires a total heating duty of 6.3 MW. After distilling the solvent, the model assumes that hexanoic and octanoic acids are separated in a second distillation column with a volume of 240 ft^3^ that requires a total heating duty of 0.75 MW.

After the liquid–liquid extraction, the aqueous phase is fed to biogas-producing anaerobic digesters (Fig. 7). Anaerobic digestion of lignocellulosic stillage [67] and acid-digested stillage [68] for biogas production has been demonstrated by others. The mass flow rate of COD to the anaerobic digesters, including stillage, lignin, and biosolids, is 21,000 kg h^−1^, resulting in biogas production of 16,600 kg h^−1^ (compared to 21,900 kg h^−1^ if the stillage is used directly as in the NREL TEA) [2]. The overall power generation from the remaining organics after MCFA removal is reduced from 41.0 MW to 38.0 MW. The reduction in overall power generation is small because lignin contributes the majority of COD to the anaerobic digesters.

As a result of the additional heating demands for the MCFA distillation columns, the net electricity that can be sold decreased from 13.7 to 3.8 MW (Table 3). In addition, the capital costs associated with the stillage fermentation reactor, liquid–liquid extraction, and distillation columns increased the total capital investment from $423 million to $441 million. The additional chemical costs for KOH and 2-octanol added annual operating costs of $14 and $8.3 million, respectively. However, the MCFA products increased revenue by $57 million ($7.5 million from octanoic acid, and $47.5 million from hexanoic acid). Based on a 30-year cash flow with a 10% internal rate of return, the minimum ethanol selling price was determined to be $1.76 per gallon ($2.68 per gallon gasoline-equivalent) (Additional file 7). This is 18% lower than the $2.15 per gallon for when electricity is generated as the only co-product to ethanol [2].

**Table 3:** Summary of technoeconomic analysis for co-production of ethanol, electricity, and MCFAs in a lignocellulosic biorefinery

Description	Size	No.	Installation factor	Total capital and installation cost
Additional capital expenses for MCFA production				
Mixed culture fermentation reactors	4 MG	4	1.0^a^	$7,540,000
Mixed culture fermentation agitators	30 hp	4	1.5	$317,000
Mixed culture fermentation feed pumps	2500 gpm	4	1.0^a^	$227,000
Caustic feed system	300 gph	4	1.0^a^	$29,800
Liquid–liquid extraction	4700 ft^3^	2	2.4	$860,000
Solvent feed system	50 gpm	1	2.4	$29,200
Distillation column 1 (solvent recovery)	630 ft^3^	1	2.4	$1,420,000
Distillation column 2 (MCFA separation)	240 ft^3^	1	2.4	$797,000

Description	Cost per ton	Annual operating expense		
Additional operating expenses for MCFA production				
KOH	$866	$14,000,000		
2-Octanol	$1402	$9,660,000		

Description	Production	Annual revenue		
Additional revenue for MCFA production				
Electricity	3759 kW	$1,810,000		
HA	1877 kg h^−1^	$47,450,000		
OA	169 kg h^−1^	$7,490,000		

Description	Ethanol–electricity co-production^b^	Ethanol–electricity–MCFA co-production		
Comparison of ethanol–electricity co-production to ethanol–electricity–MCFA co-production				
Total co-product revenue	$6,600,000	$56,800,000		
Total capital investment	$422,900,000	$441,200,000		
MESP	$2.15	$1.76		
MESP (gasoline equivalents)	$3.27	$2.68		

Capital expenses, operating expenses, and co-product revenues were used to update the NREL TEA cost model. Additional information is provided in Additional file 3

^a^Installation is included in the equipment quotes obtained from Humbird et al. [2]

^b^These values were obtained from Humbird et al. [2]

## Discussion

Our work illustrates the potential of using microbial communities to convert stillage into valuable co-products. In the stillage-fed bioreactor, productivities of hexanoic (2.6 ± 0.3 g L^−1^ day^−1^) and octanoic (0.27 ± 0.04 g L^−1^ day^−1^) acids were sustained for 214 days with titers at 66 ± 8.2 and 97 ± 15% of their solubility in water, respectively. These productivities are consistent with other studies investigating the conversion of organic substrates derived from lignocellulosic materials or ethanol production wastes to MCFAs (Additional file 8: Table S17). Our system is unique, however, in that the primary carbohydrate consumed is xylose and the stillage has already been depleted of a large portion of fermentable sugars and the ethanol that others have used to produce MFCA. While we are proposing the co-production of ethanol and MCFAs in this study, recent work has also explored production of MCFA as the main product of a lignocellulosic biorefinery. In work performed by Nelson et al., *Megasphaera* consumed glucose in lignocellulosic hydrolysate to generate hexanoic acid, but xylose was not consumed [16]. The microbial community like the one presented in this study could be utilized to convert the remaining xylose to MCFAs.

The simplicity of the microbial community enriched in this study positions it well as a model community for MCFA production. Others have shown enrichments containing OTUs related to primary sugar fermenters, such as *Lactobacillus*, and OTUs related to *Clostridia* that may be involved in converting intermediate fermentation products to MCFA [13–15, 17, 18, 47, 69]. In our microbial community, at Day 252, only 10 OTUs are present at greater than 1% relative abundance, and these OTUs make up 89.3% of the total OTUs (Additional file 5: Figure S3). The statistical analyses indicate that *Pseudoramibacter* and *Lactobacillus* are co-enriched, and their abundance correlates with higher MCFA production. We, therefore, propose that *Lactobacillus* converts xylose to lactate and acetate by heterofermentation, and the lactate is elongated to MCFAs by *Pseudoramibacter*. While 16S rRNA gene sequencing allows for the phylogeny of abundant organisms to be estimated, the function of community members should be investigated further utilizing metagenomic approaches. Due to the simplicity of the microbial community obtained in this study, this microbiome is well positioned for further investigation with metagenomic tools. Furthermore, its simplicity makes this a candidate microbiome for simulation with synthetic communities in the future. Of the OTUs that became enriched in the reactor, only *Roseburia* (denovo27808) and *Pseudoramibacter* (denovo6337) emerged as likely MCFA-producing bacteria. While *Pseudoramibacter* have been shown to produce MCFAs [66], to our knowledge, the ability of *Roseburia* to produce MCFAs has not been studied.

The TEA shows that even at the modest productivities of hexanoic and octanoic acids obtained in this study, MCFAs produced from ethanol stillage could improve the economic feasibility of lignocellulosic biorefining if the productivity can be maintained at industrial scale. Improvements in the overall conversion of stillage COD to MCFAs and production of a higher proportion of octanoic acid would further increase the revenue that can be generated by this strategy. Increasing MCFA product specificity towards octanoic acid is an ongoing area of research. One strategy to increase octanoic acid production is to utilize pertractive extraction of MCFAs to reduce product inhibition, as has been performed in past studies [13, 14]. Recent work has also shown that increasing the ratio of ethanol to acetate increases selectivity of octanoic acid production [13]. The model of increasing the ratio of reduced electron donors to acetate suggests that, in the absence of ethanol, increasing the production of lactate as a fermentation intermediate (rather than acetate) could further drive octanoic acid production.

The economy of co-producing MCFAs may also be affected by upstream biomass processing (i.e., the conversion of plant polymers to their constituent monomer units) and ethanol fermentation. For example, utilization of xylose by industrial yeast strains, such as *S. cerevisiae*, is limited [20], although attempts to improve pentose utilization by ethanol producers is an area of intense research activity [70]. Even though the *S. cerevisiae* Y128 strain used in this study was engineered for improved xylose utilization, it only consumed 47% of the xylose available in the switchgrass hydrolysate. Future ethanologenic organisms used in a lignocellulosic biorefinery may leave less xylose available for MCFA production. However, given the higher price of MCFAs compared to ethanol, decreasing xylose consumption by the ethanologenic organism may actually result in an improved economy of the lignocellulosic biorefinery.

Another simple opportunity for improving the economic potential of co-producing MCFAs is utilizing sodium hydroxide for pH control, instead of KOH, as sodium hydroxide is roughly one-sixth the cost of KOH. In our current model, the cost of KOH (Table 3) is a major expense. Alternatives to controlling pH with chemicals, such as electrolytic extraction which both controls the pH and extracts the acid products [17], should also be explored further.

## Conclusion

In this study, we tested the hypothesis that microbial communities could be used to produce valuable compounds from lignocellulosic stillage. We developed conditions for sustained MCFA production by an anaerobic microbiome that uses stillage produced during lignocellulosic biorefining. By fermenting switchgrass stillage, we maintained productivities of hexanoic and octanoic acids of 2.6 ± 0.3 and 0.27 ± 0.04 g L^−1^ day^−1^, respectively. To our knowledge, this is the first demonstration of MCFA production with xylose and other organics in lignocellulosic ethanol stillage as the primary substrates. The MCFA-producing microbial community was derived from a diverse wastewater treatment ecosystem, but over time it became enriched with OTUs representing only five genera, including members of the Firmicutes phylum (*Lactobacillus*, *Roseburia*, and *Pseudoramibacter*) and of the Actinobacteria phylum (*Olsenella* and *Atopobium*). *Pseudoramibacter* are *Clostridia* related to known MCFA-producing organisms, some of which have been shown to produce hexanoic and octanoic acids [66].

A TEA, based on an update to an industry-accepted model, shows that, at the productivity of MCFAs achieved in this study, valorizing lignocellulosic ethanol stillage to MCFAs could improve the economic sustainability of a biorefinery. For example, using the MCFA production experimentally observed, if 16% of the COD remaining in stillage is converted to hexanoic acid and 1.7% is converted to octanoic acid, the minimum ethanol selling price could be reduced by 18%, from $2.15 to $1.76 gal^−1^. Optimization of microbiome MCFA productivities, MCFA extraction, solvent recovery and selection of the ethanologenic organism may contribute further to improving the economy of the lignocellulosic biorefinery.

## Additional files

**Additional file 1.** Chemical analyses of stillage production and mixed culture fermentation reactor. **Table S1**. Results from two fermentations of switchgrass hydrolysate with Saccharomyces cerevisiae Y128. **Table S2.** Soluble COD results for fermented switchgrass hydrolysate. **Table S3.** Summary of organic components in switchgrass stillage and the mixed culture fermentation reactor. **Table S4.** Total soluble COD in switchgrass stillage and mixed culture fermentation reactor. **Table S5.** Components in stillage and mixed culture fermentation reactor samples measured by GC-MS and HPLC analyses. **Table S6.** Total soluble carbohydrates measured in stillage and mixed culture fermentation reactor with the anthrone method. **Table S7**. Total soluble proteins in stillage and mixed culture fermentation reactor measured with the BCA assay. **Table S8.** Summary of aromatic compounds in stillage.

**Additional file 2.** Rarefied tables of operational taxonomic units (OTUs) and classification with Silva. **Table S9.** OTU table for 6d experiments testing different pH conditions. **Table S10.** OTU table for 252 days of reactor operation at pH 5.5.

**Additional file 3.** Data used for development of the technoeconomic analysis (TEA).** Table S11.** Summary of process modeling for liquid-liquid extraction, solvent distillation, and MCFA separation with ASPEN**. Table S12.** Summary of capitol expenses added to the NREL TEA**. Table S13.** Summary of operating expenses and additional product revenues added to NREL TEA. **Table S14.** 30 year cash flow from NREL TEA updated to include MCFA production expenses and revenues. **Table S15.** Calculations for additional capital expenses. **Table S16.** Chemical costs for octanoic acid, hexanoic acid and 2-octanol obtained from Zauba.

**Additional file 4.** Chemical analysis for mixed culture fermentations after 6 days under different pH conditions.

**Additional file 5.** Heat map of most abundant OTUs in the initial mixed culture fermentation experiments under different pH conditions.

**Additional file 6.** Heat map of 100 most abundant OTUs from 252 days of mixed culture fermentation reactor operations.

**Additional file 7.** Summary of Technoecomoic Analysis (Output from NREL TEA Model).

**Additional file 8: Table S17.** Comparison of hexanoic (C6) and octanoic (C8) acid productivities and titers for this study and other studies utilizing cellulosic or ethanol-production derived substrates.
