# Supplementary figures and images for: Increasing the economic value of lignocellulosic stillage through medium-chain fatty acid production
Source: Biotechnol Biofuels. 2018 Jul 19;11:200. doi: 10.1186/s13068-018-1193-x (PMC6052542; doi:10.1186/s13068-018-1193-x)

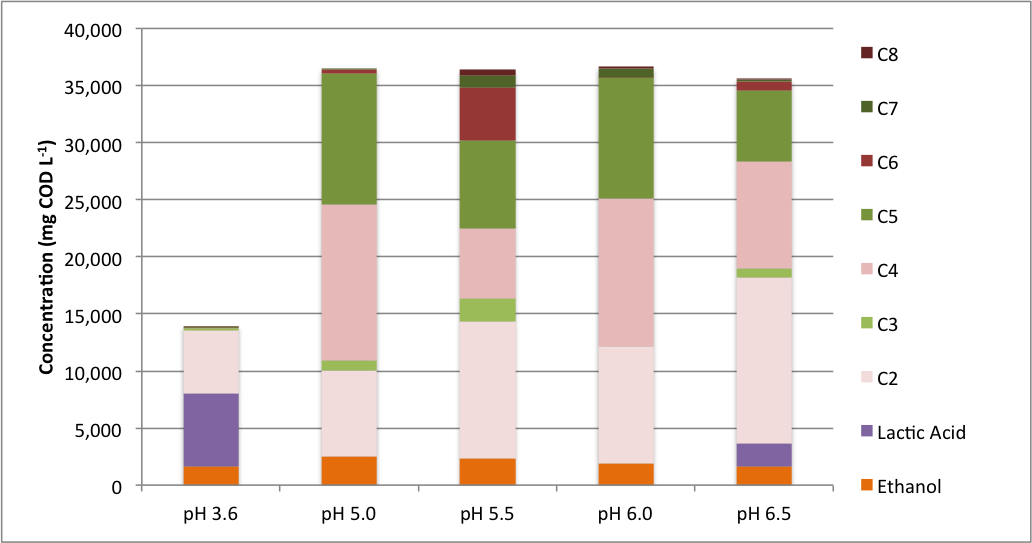

Supplement: Supplementary file 4 — Additional file 4. Chemical analysis for mixed culture fermentations after 6 days under different pH conditions. [file 13068_2018_1193_MOESM4_ESM.png]

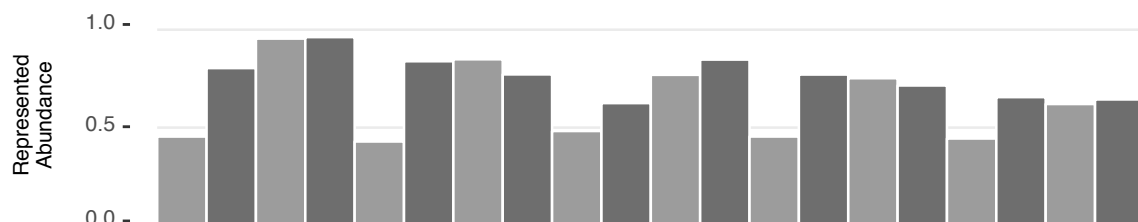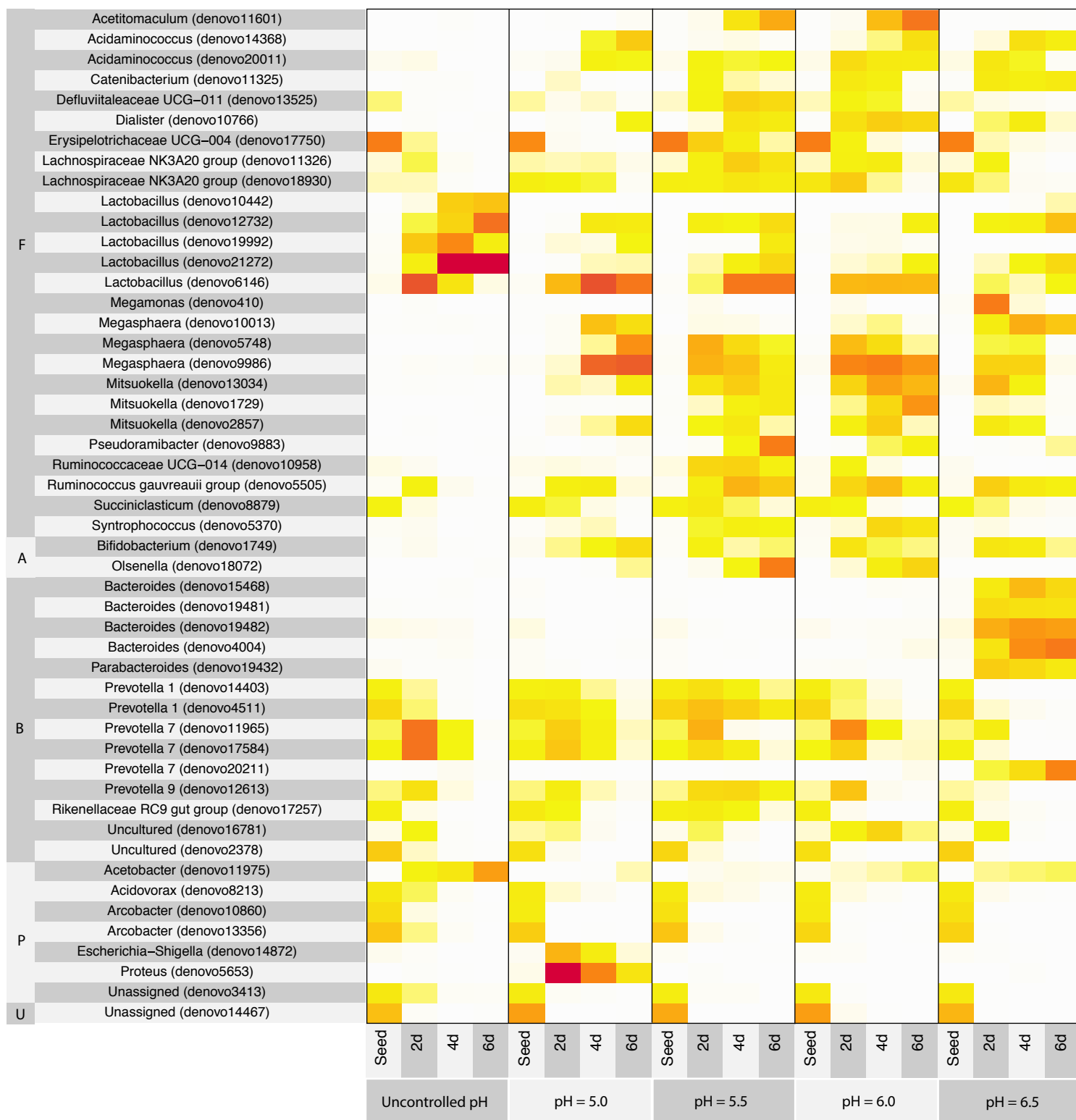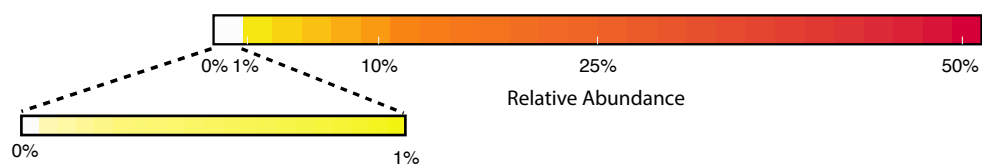

Supplement: Supplementary file 5 — Additional file 5. Heat map of most abundant OTUs in the initial mixed culture fermentation experiments under different pH conditions. [file 13068_2018_1193_MOESM5_ESM.pdf]

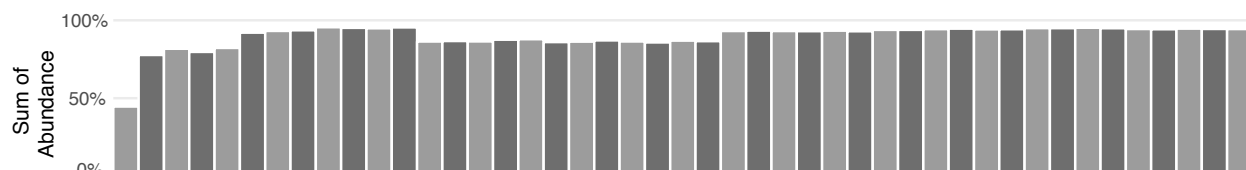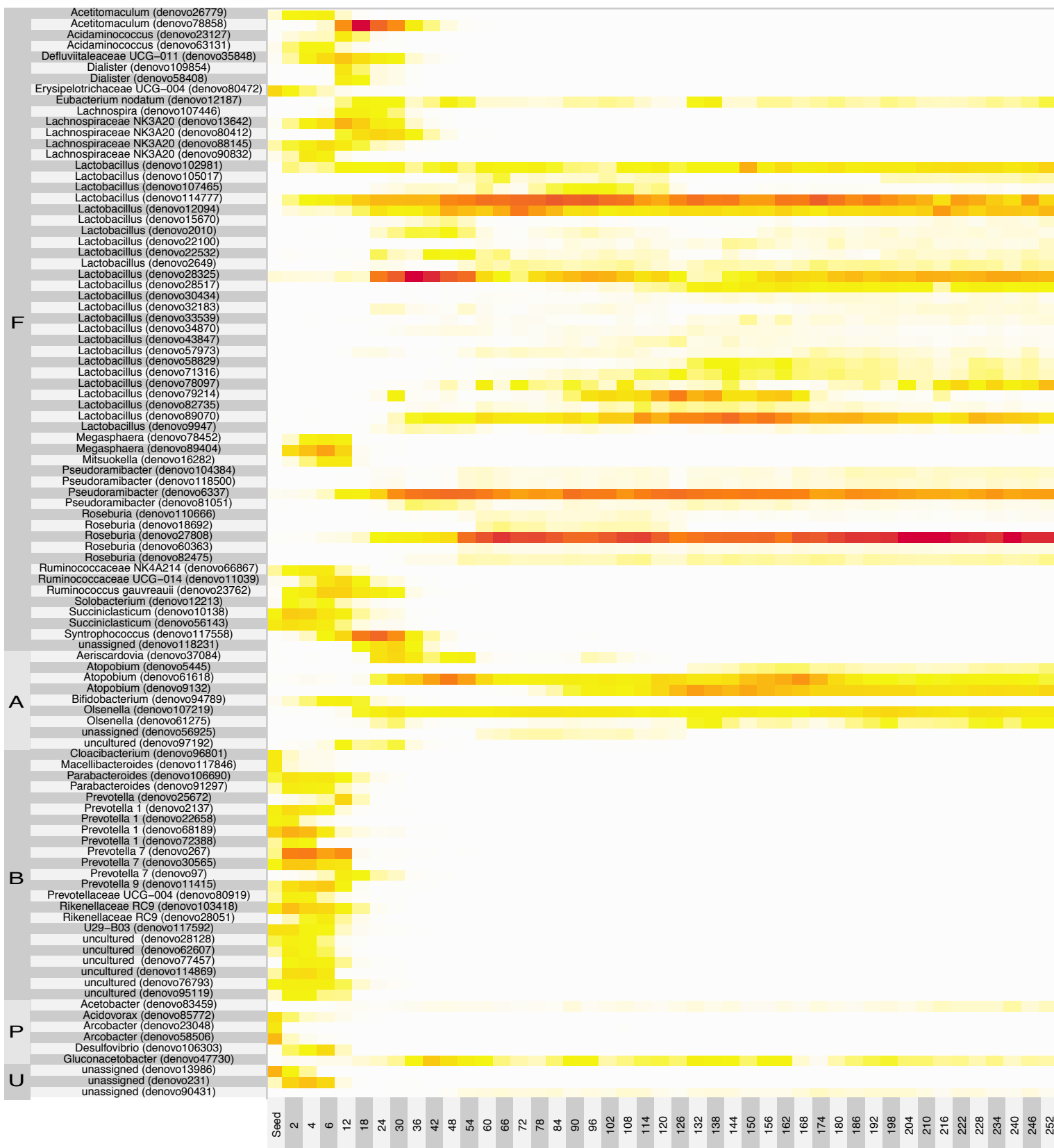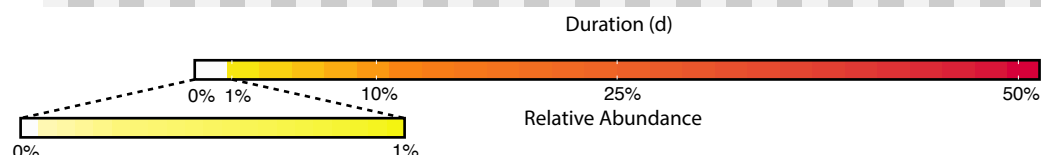

Supplement: Supplementary file 6 — Additional file 6. Heat map of 100 most abundant OTUs from 252 days of mixed culture fermentation reactor operations. [file 13068_2018_1193_MOESM6_ESM.pdf]
