# Supplementary material for: Increasing the economic value of lignocellulosic stillage through medium-chain fatty acid production
Source: Biotechnol Biofuels. 2018 Jul 19;11:200. doi: 10.1186/s13068-018-1193-x (PMC6052542; doi:10.1186/s13068-018-1193-x)
Supplement: Supplementary file 7 — Additional file 7. Summary of Technoecomoic Analysis (Output from NREL TEA Model). [file 13068_2018_1193_MOESM7_ESM.pdf]

Ethanol Production Process Engineering Analysis

Corn Stover Design Report Case: 2012 model DW1102A
Dilute Acid Pretreatment with Enzymatic Hydrolysis and Co-Fermentation
All Values in 2007$

Minimum Ethanol Selling Price (MESP): **$1.76 /gal**

Gasoline-Equivalent MESP:	**$2.68**	**/gal gasoline equivalent**
Contributions: Feedstock	**$0.74**	**/gal**
Enzymes	**$0.34**	**/gal**
Non-Enzyme Conversion	**$0.68**	**/gal**
Ethanol Production	61.0	MMgal/yr (Ethanol at 68 °F)
Ethanol Yield	79.0	gal / dry U.S. ton feedstock
Feedstock + Handling Cost	$58.50	/dry U.S. ton
Internal Rate of Return (After-Tax)	10%	
Equity Percent of Total Investment	40%	

Capital Costs	
Pretreatment	$29,900,000
Neutralization/Conditioning	$3,000,000
Saccharification & Fermentation	$31,300,000
On-site Enzyme Production	$18,700,000
Distillation and Solids Recovery	$22,300,000
Wastewater Treatment	$60,200,000
Storage	$5,000,000
Boiler/Turbogenerator	$65,800,000
Utilities	$6,900,000
Total Installed Equipment Cost	$243,100,000
Added Direct + Indirect Costs	$198,100,000
(% of TCI)	45%
Total Capital Investment (TCI)	$441,200,000
Installed Equipment Cost/Annual Gallon	$3.98
Total Capital Investment/Annual Gallon	$7.23
Loan Rate	8.0%
Term (years)	10
Capital Charge Factor (Computed)	0.131
Denatured Fuel Production (MMgal/yr)	61.7
Denatured Fuel Min. Sales Price	$1.80
Denaturant Cost ($/gal denaturant)	$2.10
Maximum Yields (100% of Theoretical)	
Ethanol Production (MMgal/yr)	80.3
Theoretical Yield (gal/U.S. ton)	103.9
Current Yield (Actual/Theoretical)	76.0%

Manufacturing Costs (cents/gal ethanol)	
Feedstock + Handling	74.1
Sulfuric Acid	2.4
Ammonia	6.5
Glucose (enzyme production)	19.3
Other Raw Materials	51.7
Waste Disposal	2.5
Coproducts	-93.0
Fixed Costs	17.7
Capital Depreciation	22.8
Average Income Tax	12.8
Average Return on Investment	59.5

Manufacturing Costs ($/yr)	
Feedstock + Handling	$45,200,000
Sulfuric Acid	$1,500,000
Ammonia	$4,000,000
Glucose (enzyme production)	$11,800,000
Other Raw Materials	$31,600,000
Waste Disposal	$1,500,000
Net Coproducts	-$56,700,000
Fixed Costs	$10,800,000
Capital Depreciation	$13,900,000
Average Income Tax	$7,800,000
Average Return on Investment	$36,300,000

Specific Operating Conditions	
Enzyme Loading (mg/g cellulose)	20
Saccharification Time (days)	3.5
Fermentation Time (days)	1.5
Ethanol titer (wt%)	5.4%
Excess Electricity (kWh/gal)	0.4
Plant Electricity Use (kWh/gal)	5.3
Plant Water Usage (gal/gal)	5.4
