# Supplementary material for: Increasing the economic value of lignocellulosic stillage through medium-chain fatty acid production
Source: Biotechnol Biofuels. 2018 Jul 19;11:200. doi: 10.1186/s13068-018-1193-x (PMC6052542; doi:10.1186/s13068-018-1193-x)
Supplement: Supplementary file 8 — Additional file 8: Table S17. Comparison of hexanoic (C6) and octanoic (C8) acid productivities and titers for this study and other studies utilizing cellulosic or ethanol-production derived substrates. [file 13068_2018_1193_MOESM8_ESM.pdf]

Additional File 8

Increasing the economic value of lignocellulosic stillage through medium through medium-chain fatty acid production

Matthew J. Scarborough,[1,2] Griffin Lynch,[1,3] Mitch Dickson,[1,3] Mick McGee,[1] Timothy J. Donohue,[1,4] and Daniel R. Noguera[1,2]*

Table S17: Comparison of hexanoic (C6) and octanoic (C8) acid productivities and titers for this study and other studies utilizing cellulosic or ethanol-production derived substrates

Substrate	Inoculum/ Organism	SRT (d)	pH	Maximum Productivity (g COD L^{-1} hr^{-1})		Maximum Titer (g COD L^{-1})		Reference
				C6	C8	C6	C8	
Switchgrass Stillage	Wastewater Acid Digester Sludge	6.0	5.5	0.13	0.017	18.7	2.39	This Study
Diluted Acid Treated Corn Stover Hydrolysate	*M. elsdenii*	7.1[1]	6.5[2]	0.17	NR	28.6	NR	[1]
Mechanically Refined Corn Stover Hydrolysate	*M. elsdenii*	7.1[1]	6.5[2]	0.18	NR	30.0	NR	[1]
Wheat Bioethanol Beer and Stillage	Enriched MCFA Producing Community	7.5	5.5	0.10	0.043	17.8	7.81	[2]
Corn Beer	Reactor Microbiome	12 to 15	5.5	0.19	Trace	NR	Trace	[3]
Corn Beer	Reactor Microbiome	15.5 to 26.3[3]	5.5	0.31	Trace	14.0	Trace	[4]
Acid Pre-treated Willow	Silage, Rumen, and Compost Mixture	21[1]	NR	NR	NR	66.0	NR	[5]
Ethanol, Alfalfa and Switchgrass	*C. Kluyveri* and Rumen Microbiome	2[1]	6.8[2]	0.13	NR	13.4	NR	[6]
Ethanol and Brewer's Spent Grain	Wastewater Activated Sludge	20[1]	7	NR	NR	0.80	NR	[7]

1. Time represents the duration of a batch experiment
2. pH represents the starting pH of a batch experiment without pH control
3. This study utilized 2-bromoethanesulfonate to inhibit methanogenesis

References:

1. Nelson R, Peterson D, Karp E, Beckham G, Salvachúa D. Mixed Carboxylic Acid Production by Megasphaera elsdenii from Glucose and Lignocellulosic Hydrolysate. Fermentation. 2017,3:10.
2. Andersen SJ, De Groof V, Khor WC, Roume H, Props R, Coma M, Rabaey K. A Clostridium Group IV Species Dominates and Suppresses a Mixed Culture Fermentation by Tolerance to Medium Chain Fatty Acids Products. Front Bioeng Biotechnol. 2017,5:8.
3. Agler MT, Spirito CM, Usack JG, Werner JJ, Angenent LT. Chain elongation with reactor microbiomes: upgrading dilute ethanol to medium-chain carboxylates. Energy Environ Sci. 2012,5:8189.
4. Ge S, Usack JG, Spirito CM, Angenent LT. Long-Term n-Caproic Acid Production from Yeast-Fermentation Beer in an Anaerobic Bioreactor with Continuous Product Extraction. Environ Sci Technol. 2015,49:8012-21.

5. Xiong B, Richard TL, Kumar M. Integrated acidogenic digestion and carboxylic acid separation by nanofiltration membranes for the lignocellulosic carboxylate platform. J Membr Sci. 2015,489:275-83.
6. Weimer PJ, Nerdahl M, Brandl DJ. Production of medium-chain volatile fatty acids by mixed ruminal microorganisms is enhanced by ethanol in co-culture with Clostridium kluyveri. Bioresour Technol. 2015,175:97-101.
7. Liang S, Wan C. Carboxylic acid production from brewer's spent grain via mixed culture fermentation. Bioresour Technol. 2015,182:179-83.
